# Sensory, Motor and Intrinsic Mechanisms of Thalamic Activity related to Organic and Psychogenic Dystonia

**DOI:** 10.4172/2161-0460.1000324

**Published:** 2017-05-15

**Authors:** K Kobayashi, JH Chien, JH Kim, FA Lenz

**Affiliations:** 1 Departments of Neurosurgery and Neurology Johns Hopkins Hospital, Baltimore, MD, USA; 2 Division of Neurosurgery, Department of Neurological Surgery, Nihon University School of Medicine, Tokyo, Japan; 3 Department of Neurosurgery, Korea University Guro Hospital, Seoul, Korea

**Keywords:** Dystonia, Thalamus, Single neuron analysis, Burst firing, Sensorimotor

## Abstract

The thalamus is a critical module in the circuit which has been associated with movement disorders including dystonia. This circuit extends from cortex to striatum to pallidum to the thalamic nucleus Ventral Lateral anterior (VLa) to cortex and can be studied by activity recorded during thalamic stereotactic surgery for the treatment of dystonia. Neuronal recordings in the VLa nucleus show low frequency modulation of firing that is correlated with and leads the low frequency modulation of EMG activity; this EMG activity is characteristic of dystonia. Immediately posterior is the Ventral Lateral posterior (VLp) nucleus which, in controls (patients with tremor or chronic pain), is characterized by deep sensory cells which fire at short latency in response to movement of a single joint or to stimulation of deep structures, such as muscles, tendons and joints. In patients with dystonia, neurons with this sensory activity are much more common than in controls and single neurons often respond to movement of multiple joints. In controls operated for the treatment of tremor or chronic pain many neurons in both nuclei are activated during active or involuntary joint movements, such as tremor or dystonia. The active joint movement related to the firing of a cell is usually in the opposite direction to the passive joint movement which causes that cell to fire. This linkage of active or involuntary and passive joint movement is unfocussed in dystonia. The involuntary dystonic joint movement best correlated with firing of a neuron may not activate the neuron when it occurs as a passive movement, while multiple other passive movements will activate the neuron. These linkages may explain the overflow of isolated voluntary activity to multiple other muscles that is seen in dystonia. The activity of either nucleus may have a critical role in dystonia since their disruption by stimulation or lesioning can decrease dystonia.

Dystonia is a movement disorder characterized by sustained muscle contractions leading to twisting repetitive movements and abnormal postures, and is characterized by abnormal forebrain activity. For example, thalamic activity in patients with organic and psychogenic dystonia is different from that in control patients with tremor or chronic pain [1,2]. Thalamic involvement was initially suggested by neuropathologic studies of patients with dystonia secondary to strokes which produced isolated lesions of the Basal Ganglia [3]. Patients with lesions of the structure receiving inputs to the Basal Ganglia from the cortex (striatum) and sparing the output structure from the Basal Ganglia (globus pallidus) developed dystonia. In turn, the globus pallidus sends an inhibitory connection to the thalamus, the only such connection in the primate thalamus [4]. In general terms, the thalamus participates in a circuit from cortex to striatum to pallidum to thalamus and then to frontal cortex, which may be segregated into discrete loops for motor, oculomotor, prefrontal and limbic functions [5].

The thalamus is classically viewed as a relay to cortex of multiple (extrinsic) inputs from other structures in the brain or in the periphery. These inputs are transmitted through functionally and anatomically specific nuclei in the thalamus to corresponding areas of cortex. These peripheral inputs include those arising from the dorsal column nuclei or the retina to the somatic sensory and visual cortex [6]. In the case of motor functions, the outputs of the deep cerebellar nuclei project through the VLp nucleus of the thalamus (Hassler’s Ventral Intermediate, to motor cortex, while the internal pallidum projects to the VLa (Hassler’s Ventral Oral posterior) to the premotor cortex as well as part of the motor cortex (Table 1) [7,8].

Neural activity in the motor thalamus is related to motor behaviors, both normal voluntary and pathologic involuntary movements. Movement in response visual or somatic cues is associated with increases in neuronal firing throughout the VLa and VLp in healthy monkeys [4,9,10]. and humans during the exploration of the thalamus prior to stereotactic procedures for tremor or dsytonia [11,12] Pathologic oscillatory activity in this nucleus was correlated with EMG activity in cases of tremor of several diagnoses [13–15]. Many of these cells show activity related to both active and passive movement, which were linked so that a cell will respond to the passive joint movement in one direction and active joint movement in the opposite direction [12]. This type of linkage is also found in the motor cortex and may represent a feedback circuit, which may become unstable and oscillate in some types of tremor [16,17].

In addition to activity related the relay of extrinsic inputs through the thalamus, there is activity related to intrinsic thalamic circuitry. In all mammalian species, thalamic relay neurons that relay inputs from the thalamus to the cortex, show activity characterized by burst firing (Grouped) or single spike firing with a relatively constant rate of action potentials (Non-grouped) or by both burst and single spike firing (Intermediate). Burst firing occurs when relay neurons are hyperpolarized and passive currents cause the membrane potential to return toward the resting level. During this process, an active calcium potential is deinactivated which produces a burst of sodium action potentials with a particular pattern of inter-spike intervals (ISIs) [18,19]. The burst is preceded by a long ISI and begins with a short ISI of less than 6 ms followed by progressively longer ISIs until the burst ends with an ISI of greater than 15 ms [20,21]. This pattern makes it possible to identify membrane events based upon analysis of the spike train.

We will first review the relationship between thalamic activity and dystonia including activity related to passive movements or sensory stimuli and to active movements and to the interactions between these types of movement. We will then consider the relationship between dystonia and activity related to intrinsic thalamic mechanisms. Finally, we will consider evidence that this thalamic activity is critical for dystonia based upon the results of thalamic stimulation and lesions.

Sensory inputs to the thalamic VL nuclei are reflected in the responses of relay neurons to deep sensory stimuli which activate receptors in muscle, tendons or joints [12]. In patients with dystonia deep sensory cells were significantly more common than in control patients with chronic pain or tremor, and the number of cells which responded to the movement of multiple joints was also increased. These responses to deep stimuli in VLp may be the result of sensory inputs to the thalamus from the dorsal column nuclei, spinothalamic tract or the cerebellar structures reflecting spinocerebellar tract inputs [22–26]. Responses of neurons in the VLa to deep sensory stimuli may arise from inputs from the cortical areas receiving inputs from these stimuli that project in sequence to putamen, in the striatum, and then to the pallidum and thalamus [27–29]. These inputs may be involved in abnormal reflex function in dystonia which includes decreased antagonist inhibition, contraction of muscles shorted by passive wrist flexion, and widespread muscle contraction in response to vibratory stimulation of the palm [30,31]. These abnormal functions may be related to the loss of intracortical inhibition and to increased long latency stretch reflexes evoked by joint rotation that may result from reflex arcs which traverse the motor cortex in somewhat the same way that tendon tap reflexes traverse the spinal cord [32–35].

In the VLp nucleus of patients with dystonia, there is evidence of reorganization of somatic sensory inputs. More cells with deep receptive fields are found in patients with dystonia than in control patients with tremor and chronic pain without motor abnormality. These fields were larger as evident by responses to movement of multiple joints [36]. The increase in receptive fields may be related to the increase in the size of fields in which cutaneous sensations were evoked by micro stimulation in the cutaneous core of the principle sensory nucleus of human thalamus [1]. During pathologic involuntary movements in patients with dystonia versus control subjects, the activity of neurons in the VLa is characterized by greater power in the lowest (dystonic) frequencies. Overall, this dystonia related activity is often correlated with EMG activity and phase advanced on EMG activity in muscles other than the muscle which produces the movement that leads to firing of the cell. Therefore, the sensory motor linkage is unfocussed in patients with dystonia so that sensory input from one muscle may influence activity in neurons which are related to the activity of muscles over a widespread distribution including other limbs. In addition to the changes in the influence of extrinsic sensory and motor inputs to the thalamus, there is now clear evidence that intrinsic processes in the thalamus are altered in dystonic patients.

As described above, power in the EMG spectrum of patients during dystonic movements often showed a maximum in the lowest frequency estimate of the auto power spectrum after smoothing, which corresponded to a frequency of 0.4 Hz (dystonia frequency). Based upon the distribution of the EMG across individual intervals, a signal to noise ratio (SNR) at dystonia frequency of greater than or equal to two (DF) was taken to indicate a substantial concentration of power at dystonia frequency. DF activity is seen more often for dystonia occurring during active movement than for spontaneous dystonia. Spontaneous dystonia associated with an SNR of less than two is designated as non-dystonia frequency (nDF). In VLa, spike trains during spontaneous activity were Grouped in dystonia to a greater extent than in controls. The inhibition before the burst and the rate of bursts was higher spontaneous activity of patients having organic dystonia versus health monkeys and a patient with dystonia of psychogenic origin. The duration of the inhibition is consistent with the gamma amino butyric acid receptor - type B [37]. Overall, spontaneous organic dystonia is associated with inhibition related bursting, which may result from gamma amino butyric acid type B, an observation which may have therapeutic implications for dystonia.

These results have been contrasted between patients with organic dystonia versus the patient with dystonia, which was found to be psychogenic based upon complete permanent abolition of dystonia following a short trial of physiotherapy. The disappearance of dystonia occurred years after the surgery at which thalamic recordings were carried out [2]. The dystonia frequency neuronal activity was correlated with EMG to the same extent in organic and psychogenic dystonia. The SNR at dystonia frequency in VLp was greater in organic than psychogenic dystonia and both were greater than in controls with chronic pain. The correlation of thalamic spike trains with EMG (Coherence) was significantly higher in organic dystonia but the relative phase of correlated activity was not different from zero for either etiology. The proportion of cells in the VLp responding to joint movement was lower in organic than psychogenic dystonia and there were no differences in the burst or single spike firing patterns.

To test whether any of this sensory, motor or intrinsic activity is involved in dystonic motor behavior, we examined the effect of disruption of these nuclei by micro stimulation or lesions during thalamic procedures for the treatment of dystonia [38]. In dystonic patients, micro stimulation of VLp produced ‘dystonic postures’ and simultaneous EMG activity in multiple different muscles of the contralateral arm [36]. No EMG response to stimulation was found in VLa of dystonic patients, or in either nucleus of patients with chronic pain [36,39]. In patients with tremor, stimulation of the VLp leads to decreases in the ongoing tremor, and chronic macro stimulation through implanted deep brain electrodes is a well-established treatment for essential tremor [40].

Although micro stimulation evoked muscle twitches are not found in patients with chronic pain they were produced in the monkey thalamus produced by stimulation at sites histologically confirmed in VLp but not in VLa. This difference may reflect connections of the posterior, buy not the anterior nucleus through motor cortex to the periphery [41] (also see [42]). The thalamus in monkeys is much smaller than in humans but currents of micro stimulation are comparable across species. Therefore stimulation in monkeys may activate a relatively larger volume of VLp, which may be sufficient to cause muscle twitches in monkeys but not humans. Although these results suggest that the activity in the VLa and VLp nuclei is involved in dystonia, the interruption of dystonia by small thalamic lesions more clearly demonstrates that the lesioned area and its activity are involved in the mechanism of dystonia.

Single electrolytic lesions with a volume of 30 mm^3^ in either VLa of VLp produce an immediate short term decrease in ongoing dystonic EMG activity of multiple muscles of the contralateral upper extremity [36]. Long term marked to moderate improvement in dystonia was found in 47% of patients after thalamotomy, while an improvement of greater than 50% was observed in half of patients in another study [43,44]. In the latter study lesions were large since they included multiple stages and each stage consisted of multiple lesions, each lesion with an approximate volume of 35 mm [44]. At present, stimulation of the internal pallidum bilaterally is the most common surgical approach to the treatment of medically intractable dystonia [45–47]. The results of the earlier literature of single small lesions of VLa or VLp or both, and much larger lesions suggest that thalamic dystonia related activity, somatic sensory reorganization and changes in intrinsic thalamic circuitry are involved in the mechanism of dystonia.

## Figures and Tables

**Table 1:** Abbreviations: thalamic nuclei, EMG electromyogram, ISI inter-spike interval.

Thalamic Nuclei Nomenclatures	Hirai and Jones for human and monkey [7], corresponding to Hassler [46] (also see [47]).
Pallidal relay nucleus	Ventral Lateral anterior (VLa), corresponding to Ventral Oral posterior.
Cerebellar relay nucleus	Ventral Lateral posterior (VLp), corresponding to Ventral Intermediate.
